# Insights into group-specific pattern of secondary metabolite gene cluster in *Burkholderia* genus

**DOI:** 10.3389/fmicb.2023.1302236

**Published:** 2024-01-16

**Authors:** Byeollee Kim, So-Ra Han, Hyun Lee, Tae-Jin Oh

**Affiliations:** ^1^Department of Life Science and Biochemical Engineering, Graduate School, SunMoon University, Asan, Republic of Korea; ^2^Genome-Based BioIT Convergence Institute, Asan, Republic of Korea; ^3^Division of Computer Science and Engineering, SunMoon University, Asan, Republic of Korea; ^4^Department of Pharmaceutical Engineering and Biotechnology, SunMoon University, Asan, Republic of Korea

**Keywords:** *Burkholderia* genus, comparative genomics, biosynthetic gene cluster, pattern analysis, network analysis

## Abstract

*Burkholderia* is a versatile strain that has expanded into several genera. It has been steadily reported that the genome features of *Burkholderia* exhibit activities ranging from plant growth promotion to pathogenicity across various isolation areas. The objective of this study was to investigate the secondary metabolite patterns of 366 *Burkholderia* species through comparative genomics. Samples were selected based on assembly quality assessment and similarity below 80% in average nucleotide identity. Duplicate samples were excluded. Samples were divided into two groups using FastANI analysis. Group A included *B. pseudomallei* complex. Group B included *B. cepacia* complex. The limitations of MLST were proposed. The detection of genes was performed, including environmental and virulence-related genes. In the pan-genome analysis, each complex possessed a similar pattern of cluster for orthologous groups. Group A (n = 185) had 14,066 cloud genes, 2,465 shell genes, 682 soft-core genes, and 2,553 strict-core genes. Group B (n = 181) had 39,867 cloud genes, 4,986 shell genes, 324 soft-core genes, 222 core genes, and 2,949 strict-core genes. AntiSMASH was employed to analyze the biosynthetic gene cluster (BGC). The results were then utilized for network analysis using BiG-SCAPE and CORASON. Principal component analysis was conducted and a table was constructed using the results obtained from antiSMASH. The results were divided into Group A and Group B. We expected the various species to show similar patterns of secondary metabolite gene clusters. For in-depth analysis, a network analysis of secondary metabolite gene clusters was conducted, exemplified by BiG-SCAPE analysis. Depending on the species and complex, *Burkholderia* possessed several kinds of siderophore. Among them, ornibactin was possessed in most *Burkholderia* and was clustered into 4,062 clans. There was a similar pattern of gene clusters depending on the species. NRPS_04014 belonged to siderophore BGCs including ornibactin and indigoidine. However, it was observed that each family included a similar species. This suggests that, besides siderophores being species-specific, the ornibactin gene cluster itself might also be species-specific. The results suggest that siderophores are associated with environmental adaptation, possessing a similar pattern of siderophore gene clusters among species, which could provide another perspective on species-specific environmental adaptation mechanisms.

## 1 Introduction

*Burkholderia* classified in the phylum *Proteobacteria* have been isolated from various sources worldwide (Radua et al., 2000; Levy et al., 2008; Hall et al., 2015; Peddayelachagiri et al., 2016), including cystic fibrosis patients (Medina-Pascual et al., 2015) and environmental sources such as soil (Hall et al., 2015), rice (Luo et al., 2007), and lichen (Han et al., 2016). The genus *Burkholderia* was named in 1992. It initially consisted of seven pathogenic strains affecting humans, animals, and plants (Yabuuchi et al., 1992). It has been categorized into two major groups: *Burkholderia cepacia* complex (BCC) and environmental *Burkholderia* (Compant et al., 2008; Sousa et al., 2011). However, some studies have focused on the beneficial effects of *Burkholderia* in plants, leading to its reclassification into new genera such as *Paraburkholderia* or *Caballeronia* (Kaur et al., 2017). Expansion of the *Burkholderia* genus is ongoing (Euzéby, 1997; Estrada-de Los Santos et al., 2018). The multi-genus complex that includes *Paraburkholderia* and *Caballeronia* is referred to as *B. sensu lato* which encompasses *Robbsia*, *Pararobbsia*, *Burkholderia*, *Trinickia*, and *Mycetohabitans*. Species reclassified within *Burkholderia* now fall under *B. sensu lato*, which also includes *Burkholderia*. *B. sensu stricto* encompasses BCC, *Burkholderia pseudomallei* complex (BPC), and rice pathogenic *Burkholderia*.

Recent reports of *Burkholderia* have highlighted the use of genome-based classification (Jin et al., 2020), genomic diversity analysis (Gee et al., 2021), and pan-genome analysis (Lee et al., 2021). After the report on the genome-based classification of BCC, the taxonomic position of the inherent Taxon K has been reevaluated (Vanlaere et al., 2009). Recent reports have focused on the reclassification and comparison analysis of genomics due to the registration of various genomes in databases. It is possible to study genome-based classification as well as secondary metabolites (Bach et al., 2022a) and evolutionary comparisons (Yu et al., 2006).

In the multi-genus complex known as *Burkholderia sensu lato*, antimicrobial compounds have been reported (Petrova and Mahenthiralingam, 2022; Rodríguez-Cisneros et al., 2023). This study primarily compiles the antimicrobial compounds identified in *Burkholderia*. Research in the field of discovery has progressed, including studies of new antimicrobial secondary metabolites associated with *Burkholderia*, and antimicrobial secondary metabolites are related to taxonomy (Depoorter et al., 2016; Deng et al., 2023). In *Burkholderia*, a wide range of natural products have been reported, including antimicrobial compounds and metabolites related to biocontrol and agriculture (Sulochana et al., 2014; Esmaeel et al., 2016, 2018; Kunakom and Eustáquio, 2019). For example, the antimicrobial compound bactobolin has been identified in *Burkholderia thailandensis* E264 (Seyedsayamdost et al., 2010). In addition, siderophores known for their roles in plant growth promotion have been detected in pathogenic *Burkholderia*.

Despite ongoing genomic and metabolite studies on *Burkholderia*, studies on *B. sensu stricto* have been limited and treated as a secondary aspect within the broader context of *B. sensu lato* study. In this study, we aimed to understand the characteristics of BCC and BPC complexes within *B. sensu stricto*. Different from previous studies focusing on the prevalence of BGCs according to individual genera within *B. sensu lato* (Mullins and Mahenthiralingam, 2021), we conducted a more detailed analysis of BGCs within *Burkholderia*, considering species variations. Furthermore, we aimed to uncover the interrelationships among different species and metabolites through network analysis.

## 2 Materials and methods

### 2.1 Preparation of *Burkholderia* genome

Complete genome sequences of 458 *Burkholderia* available in the NCBI genome database were downloaded on 17 February 2023 (Benson et al., 2017). Duplicated assemblies were removed prior to analysis. Information about strain isolation was searched in the database at NCBI (Barrett et al., 2012) and ENA (Cummins et al., 2022). Samples were categorized into three groups: none (unknown sample), PATH (pathological sample), and ENV (environmental sample) based on isolation information. We confirmed validated *Burkholderia* species in the LPSN database (Parte et al., 2020). The assembly quality of these genomes was determined using CheckM (Parks et al., 2015) and QUAST v5.2.0 (Gurevich et al., 2013) with default parameters. Assemblies were selected based on criteria of at least 90% completeness, less than 10% contamination, and a calculated value of completeness minus 5 times contamination greater than 50%. For further analysis, all genomes were annotated with prokka using default parameters (Seemann, 2014).

### 2.2 Phylogenomic analysis

Average Nucleotide Identity (ANI) was analyzed using FastANI (Jain et al., 2018) with default settings (K-mer size = 16, threads count for parallel execution = 1, fragment length default = 3,000). We analyzed multiple genomes using a genome list with command options of “—ql” and “--al.” For visualization, we used “-matrix” command. For subsequent analysis, genomes with over 80% similarity were selected using FastANI. We visualized ANI results as a heatmap using ggplot2 package in R (Wickham, 2016). Referring to the study of Wallner et al. (2019), we analyzed genes listed as environmental-related genes (*nthAB*, *oxd*, *lipA*, *faeB*, *prnA-D*, and *uxaAB*) and human virulence genes (*clab*, *adhA*, *esnR*, *amil*, *ccil*, *cciR*, *opcl*, *kdgR*, *baiE*, *taruX*, *xsc*, *telA*, *terCEF*, *narG-J*, *narLM*, *narX*, and *lxa*) using blastp (Camacho et al., 2009).

### 2.3 Pan-genome analysis

Pan-genome and core-genome were analyzed using PEPPAN (Zhou et al., 2020). Prokka results in “.gff” file format were compiled for pan-genome analysis. PEPPAN uses a reference-based approach to generate an alignment for each gene group, which is then used to reconstruct a neighbor-joining gene tree using RapidNJ. In the case of PEPPAN results, “.gff” files were created as results. We generated them using “PEPPAN_parser.” Results of the “PEPPAN_parser” displayed summaries, curve information, and gene presence-absence. We used curve information for visualization with a law model (Tettelin et al., 2008). All these processes were performed for group A and group B separately. The pan-genome value, referred to as gamma, was calculated using the Heaps’ law model (Lü et al., 2010). Values of pan-genome and core-genome were calculated as alpha using the Power law model (Tettelin et al., 2008). We analyzed orthologues of core-genes and pan-genes using the web version of EGGNOG-mapper v2.1.9 (Cantalapiedra et al., 2021). Clusters of Orthologous Groups (COGs) results were visualized and preprocessed using R. We excluded no-detected COGs and duplicated COGs.

### 2.4 Secondary metabolite gene cluster analysis

Gene cluster analysis was conducted using the local version of antiSMASH (Blin et al., 2019) with command options of “--mibig.” To visualize the results of antiSMASH, we conducted PCA based on BGC composition. We measured covariance and used scaled data for PCA analysis. These analyses obtained eigenvalues and eigenvectors using scikit-learn in Python. We visualized PCA data using Matplotlib (Hunter, 2007).

### 2.5 Network analysis in BGCs

Advanced analysis was performed using antiSMASH with the “--cc-mibig” command for comparison with the MIBiG database (Terlouw et al., 2023). For a more detailed analysis of BGCs, output results from antiSMASH were subjected to network and phylogeny analyses using BiG-SCAPE and CORASON (Navarro-Muñoz et al., 2020). We collected genes involved in BGCs already known and present in antiSMASH results. These genes were confirmed using blastp. We extracted the results of antiSMASH and information about similar gene clusters in MIBiG using Python. Gene clusters found to be similar to reference gene clusters were labeled in the network analysis. BiG-SCAPE offers advantages for conducting large-scale analyses and predicting domains using the pfam database (Mistry et al., 2021) and hmmscan from HMMER (Mistry et al., 2013). The process starts with calculating sequence similarity, followed by measuring pairwise distances between BGCs using sequence similarity. Network analysis was visualized using Cytoscape v3.10.0 (Shannon et al., 2003). Gene cluster visualization and comparison were conducted using Easyfig v2.2.5 (Sullivan et al., 2011).

### 2.6 Statistical analysis

Statistical analysis for the basic genome was performed using QUAST and tabulated. We conducted a Kruskal-Wallis test and *post hoc* Dunn test for multiple comparisons between species. Results were visualized using ggplot2. Pan-genome analysis was conducted using Heaps’ law and Power law model for each Gamma value and Kappa value to check significant differences using PEPPAN. To compare genome features, we performed statistical analysis using Mann–Whitney in R. For group comparisons, we used the Mann–Whitney test. Data were visualized using the ggbetweenstats package in R.

## 3 Results and discussion

### 3.1 Curation and reclassification of genomic dataset

To ensure data integrity, we implemented two exclusion criteria. Firstly, we removed duplicated samples sourced from NCBI. Additionally, we excluded samples that did not meet the criteria after assessing their completeness and contamination using CheckM. To accurately analyze species of *Burkholderia*, the frequent occurrence of genus expansion, as reported in *Burkholderia*, was considered (Lin et al., 2020). We excluded samples with an ANI value below 83%, following the recommendation provided in the reference of fastANI. We excluded samples based on fastANI analysis, where species with ANI values below 83% were considered inter-species. This distinction was explained by Konstantinidis and Tiedje (2005), who confirmed that an ANI value above 94% corresponded to a DNA–DNA hybridization of around 70%. Excluded samples based on these results were documented in Supplementary Table 1. Species displaying ANI values lower than 83% included *B. glumae*, *B. gladioli*, and *B. plantarii* known to be plant pathogens belonging to *B. sensu stricto* (Kim et al., 2021; Bach et al., 2022b). *B. glumae*, *B. gladioli*, and *B. plantarii* are known as rice pathogenic bacteria belonging to *B. sensu stricto*. According to Bach et al. (2022b), *B. sensu stricto* shared a low number of conserved genes with *Burkholderia* spp. and BCC.

**Figure 1:**
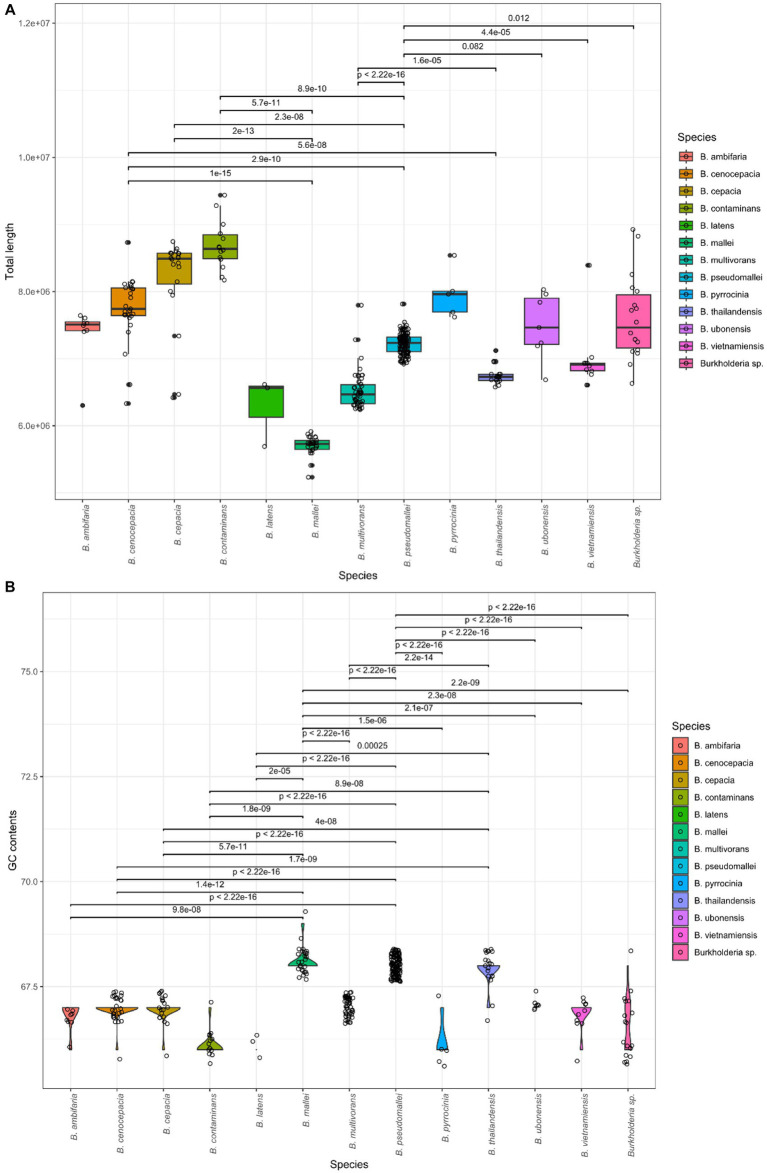
Comparison of genomic features in *Burkholderia* spp. **(A)** Average total length per species. **(B)** Average GC content per species.

A total of 366 assembly samples were processed for a subsequent study, and samples were well divided by BCC and BPC. A total of 366 strains were validated by *Burkholderia* in the LPSN database. These samples were confirmed as contigs, total length, GC contents, and N50 using QUAST. We computed statistics for each species. The results are summarized in Table 1. The information for all samples is also included in Supplementary Table 2. The average values were 2.7 contigs, with a 7.18 Gb total length, and 67.43% GC content with a mean and average N50 length of 3.62 Gb. We confirmed 30 types of species, which contained BCC and BPC (Vanlaere et al., 2008; Price et al., 2013; Beukes et al., 2017; Tuanyok et al., 2017; Depoorter et al., 2020; Hall et al., 2022; Morales-Ruíz et al., 2022). The total length and GC contents were compared for species using the Kruskal-Wallis test in R (value of *p* < 2.2e − 16; Figure 1). Genome sizes of *Burkholderia* were observed to range from a minimum of 5.2 Gb to a maximum of 9.4 Gb. GC contents ranged from 66% to 69%. Statistically significant variations of genome features were also detected among the species. In the *post hoc* Dunn test, if the value of *p* was less than or equal to alpha/2 (where alpha = 0.05), the null hypothesis was rejected. We visualized the length of the genome and GC content of species showing significant differences, along with their corresponding *p*-values as shown in Figure 1.

**Table 1:** The overview of genomic features of *Burkholderia* species analyzed in this study.

Species^*^	Complex	Groups	Contig	Total length	GC contents	N50
*Aenigmatica* (*n* = 1)	*B. cepacia* complex	B	4.00 ± 0	9,449,413 ± 0	65.76 ± 0	3,713,971 ± 0
*Ambifaria* (*n* = 8)	*B. cepacia* complex	B	4.00 ± 1.22	7365060.75 ± 408939.05	66.66 ± 0.12	2829706.75 ± 253148.89
*Anthina* (*n* = 2)	*B. cepacia* complex	B	3.50 ± 1.50	7456936.50 ± 1000436.50	66.32 ± 0.04	3,275,428 ± 189,422
*Arboris* (*n* = 1)	*B. cepacia* complex	B	3.00 ± 0	8,573,812 ± 0	66.81 ± 0	3,525,317 ± 0
*Cenocepacia* (*n* = 27)	*B. cepacia* complex	B	3.40 ± 0.95	7745151.70 ± 476253.71	66.97 ± 0.24	3534080.51 ± 1286195.86
*Cepacia* (*n* = 20)	*B. cepacia* complex	B	3.10 ± 0.88	8189106.85 ± 659640.25	66.72 ± 0.17	3646257.55 ± 735245.99
*Contaminans* (*n* = 14)	*B. cepacia* complex	B	4.71 ± 1.22	8689272.71 ± 352361.14	66.23 ± 0.19	3238472.92 ± 61459.58
*Diffusa* (*n* = 1)	*B. cepacia* complex	B	3.00 ± 0	6,857,833 ± 0	66.47 ± 0	2,619,120 ± 0
*Dolosa* (*n* = 5)	*B. cepacia* complex	B	3.00 ± 0	6366878.2 ± 85622.97	66.99 ± 0.03	3407799.8 ± 1529.98
*Humptydooensis* (*n* = 1)	*B. psedomallei* complex	A	3.00 ± 0	7,286,661 ± 0	67.14 ± 0	4,068,027 ± 0
*Lata* (*n* = 3)	*B. cepacia* complex	B	3.00 ± 0	8606041.66 ± 183289.35	66.32 ± 0.13	3560107.33 ± 131784.60
*Latens* (*n* = 3)	*B. cepacia* complex	B	3.66 ± 0.47	6289637 ± 424512.37	66.33 ± 0.04	3297008.66 ± 46764.47
*Mallei* (*n* = 27)	*B. psedomallei* complex	A	1.96 ± 0.18	5702122.44 ± 137978.69	68.45 ± 0.03	3606983.29 ± 402520.32
*Mayonis* (*n* = 2)	*B. psedomallei* complex	A	2.00 ± 0	6974222 ± 383308	66.36 ± 0.10	4,139,371 ± 300,571
*Metallica* (*n* = 1)	*B. cepacia* complex	B	3.00 ± 0	7,424,240 ± 0	67.09 ± 0	2,967,116 ± 0
*Multivorans* (*n* = 50)	*B. cepacia* complex	B	3.30 ± 0.64	6518391.70 ± 277268.32	67.12 ± 0.14	3356319.24 ± 238452.50
*Oklahomensis* (*n* = 3)	*B. psedomallei* complex	A	2.00 ± 0	7257963.33 ± 87095.28	66.97 ± 0.06	4172494.66 ± 28808.71
*Orbicola* (*n* = 1)	*B. cepacia* complex	B	3.00 ± 0	7,971,389 ± 0	66.60 ± 0	3,213,911 ± 0
*Pseudomallei* (*n* = 128)	*B. psedomallei* complex	A	2.11 ± 0.36	7226784.58 ± 150066.83	68.09 ± 0.15	4026508.67 ± 87456.08
*Pseudomultivorans* (*n* = 1)	*B. cepacia* complex	B	3.00 ± 0	7,956,789 ± 0	67.30 ± 0	4849929 ± 0
*Pyrrocinia* (*n* = 5)	*B. cepacia* complex	B	3.20 ± 0.40	7963581.20 ± 323,679.78	66.42 ± 0.10	3190692.6 ± 154980.73
*Savannae* (*n* = 2)	*B. psedomallei* complex	A	3.00 ± 0	7,254,364 ± 173,191	67.18 ± 0.13	4,153,083 ± 75,195
*Seminalis* (*n* = 2)	*B. cepacia* complex	B	3.00 ± 0	7,835,640 ± 186,696	67.16 ± 0.13	3,031,317 ± 18,819
*Burkholderia* sp. (*n* = 18)	*-*	B	3.44 ± 0.76	7607546.66 ± 607,666.25	66.68 ± 0.46	3127340.5 ± 532685.47
*Stabilis* (*n* = 2)	*B. cepacia* complex	B	3.00 ± 0	8124525.50 ± 403,421.50	66.56 ± 0.14	3241054.5 ± 77825.5
*Stagnalis* (*n* = 1)	*B. cepacia* complex	B	3.00 ± 0	7,583,807 ± 0	67.65 ± 0	3,001,569 ± 0
*Territorii* (*n* = 1)	*B. cepacia* complex	B	3.00 ± 0	6,902,370 ± 0	66.73 ± 0	2,466,714 ± 0
*Thailandensis* (*n* = 19)	*B. psedomallei* complex	A	2.15 ± 0.36	6746841.73 ± 128903.21	67.63 ± 0.10	3844628.42 ± 80,387.98
*Ubonensis* (*n* = 7)	*B. cepacia* complex	B	3.42 ± 0.49	7486224.85 ± 453586.04	67.11 ± 0.21	3105116.71 ± 511667.56
*Vietnamiensis* (*n* = 10)	*B. cepacia* complex	B	4.00 ± 1.48	7011401.40 ± 472445.79	66.85 ± 0.39	2609459.4 ± 992678.91

It is represented average values and standard deviations of genome features. The number of samples for each species is indicated by notation (such as *n* = 1).

### 3.2 Phylogenomic analysis for BCC and BPC

**Figure 2:**
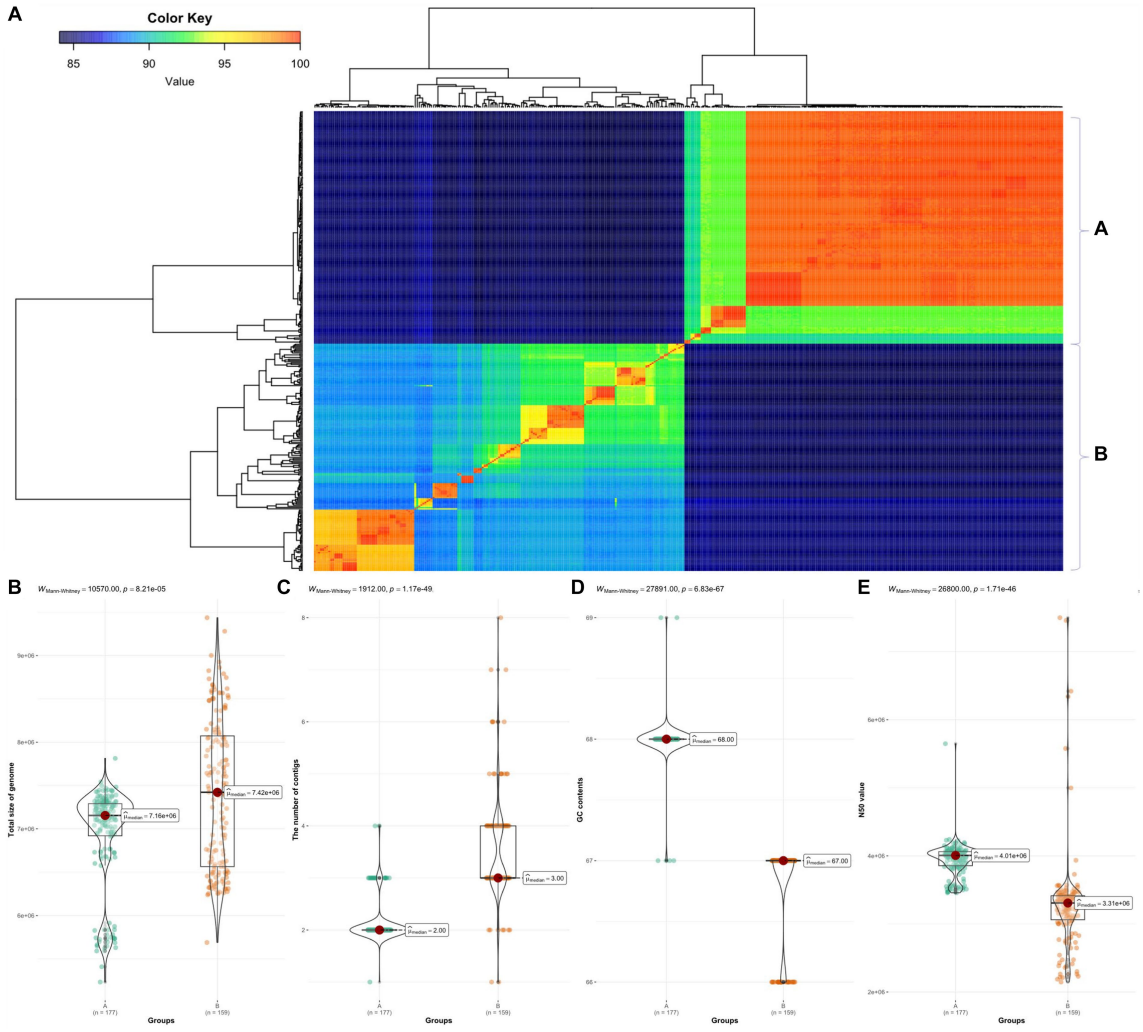
ANI in 366 *Burkholderia* spp. **(A)** ANI results using fastANI visualized using ggplot2. We divided samples into groups A and B. **(B)** Comparison of total length between group A and group B. **(C)** Comparison of contigs between group A and group B. **(D)** Comparison of GC contents between group A and group B. **(E)** Comparison of N50 between group A and group B. All comparisons were visualized using ggbetweenstats package in R.

We calculated pairwise ANI for 366 *Burkholderia* species (Figure 2A). Based on ANI analysis results, the 366 *Burkholderia* species were divided into two groups denoted as group A and group B. Group A included *B. humptydooensis*, *B. mallei*, *B. mayonis*, *B. oklahomensis*, *B. pseudomallei*, *B. savannae*, *B.* sp., and *B. thailandensis*. The group B were contained *B. aenigmatica*, *B. ambifaria*, *B. anthina*, *B. arboris*, *B. cenocepacia*, *B. cepacia*, *B. contaminans*, *B. diffusa*, *B. dolosa*, *B. lata*, *B. latens*, *B. metallica*, *B. multivorans*, *B. orbicola*, *B. pseudomultivorans*, *B. pyrrocinia*, *B. seminalis*, *B.* sp. *B. stabilis*, *B. stagnalis*, *B. territorii*, *B. ubonensis*, and *B. vietnamiensis*. The high similarity observed between *B. pseudomallei* and *B. mallei* in ANI results was consistent with merging results reported by Wallner et al. (2019). To determine differences between groups, the Mann–Whitney test was used for comparisons of contigs, length, GC contents, and N50 (Figure 2). Group B contained BCC such as *B. cepacia*, *B. multivorans*, *B. cenocepacia*, and *B. stabilis* (Lipuma, 2005). *Burkholderia* species with similarities over 99% were reclassified as similar species (Supplementary Table 3).

We visualized the data using Minimum Spanning Tree analysis based on curated MLST genes for *Burkholderia*, which included *aptD*, *gltB*, *gyrB*, *recA*, *lepA*, *phaC*, and *trpB* known in BCC, as well as *ace*, *gltB*, *gmhD*, *lepA*, *lipA*, *narK*, and *ndh* known in *B. mallei* and *B. pseudomallei*. However, no notable peculiarities or distinctive patterns were identified (Supplementary Figure 1). Following the findings of Mullins et al. (2019), who concluded the possibility of expandability due to the limitations of curated genes, we collected genes and visualized their presence using BLAST analysis (Supplementary Table 4). However, selected genes did not separate each isolation, species, or group. Genes obtained from the Uniprot database included virulence-related genes (such as adhA, kdgR, narG, narH, narX, telA, terC, xsc, and amiI) and environmental-related genes including oxd, PrnA, prnC, PrnD, and uxaA. We constructed a database with these acquired genes and confirmed them using blastp. Results did not match the separation information recorded in NCBI or ENA. However, it was important to note that confirmed genes varied by species, indicating that these genes could serve as additional factors for confirming species, addressing limitations of MLST with only BCC, *B. mallei*, and *B. pseudomallei* available in pubMLST. The distinction between pathogenic and non-pathogenic strains can be confirmed by the presence of virulence factors. Taxonomic analysis may not serve as a clear indicator of their pathogenic potential. In comparative genomics studies, it remains a challenge to determine a suitable indicator for phenotypic characteristic analysis (Eberl and Vandamme, 2016). Investigating pathogenicity and non-pathogenicity is not straightforward.

While we cannot specifically discriminate individual species, our results suggest that for group B, which comprises species belonging to BCC, it would be possible to determine whether the target species is part of the BCC or not. Genomes of *Burkholderia* used in this study displayed significant similarity in cases where samples were obtained from the same hospital, which led to time-consuming analyses. Therefore, we propose that *Burkholderia* is the most valuable sample for making a pangenome graph (Hickey et al., 2023). By clustering samples and excluding similar regions, it would be possible to create a pangenome graph for comparison not only within *Burkholderia*, but also for a broader range of *B. sensu lato* and other related microorganisms.

### 3.3 Pan-genome analysis for each group of *Burkholderia*

**Figure 3:**
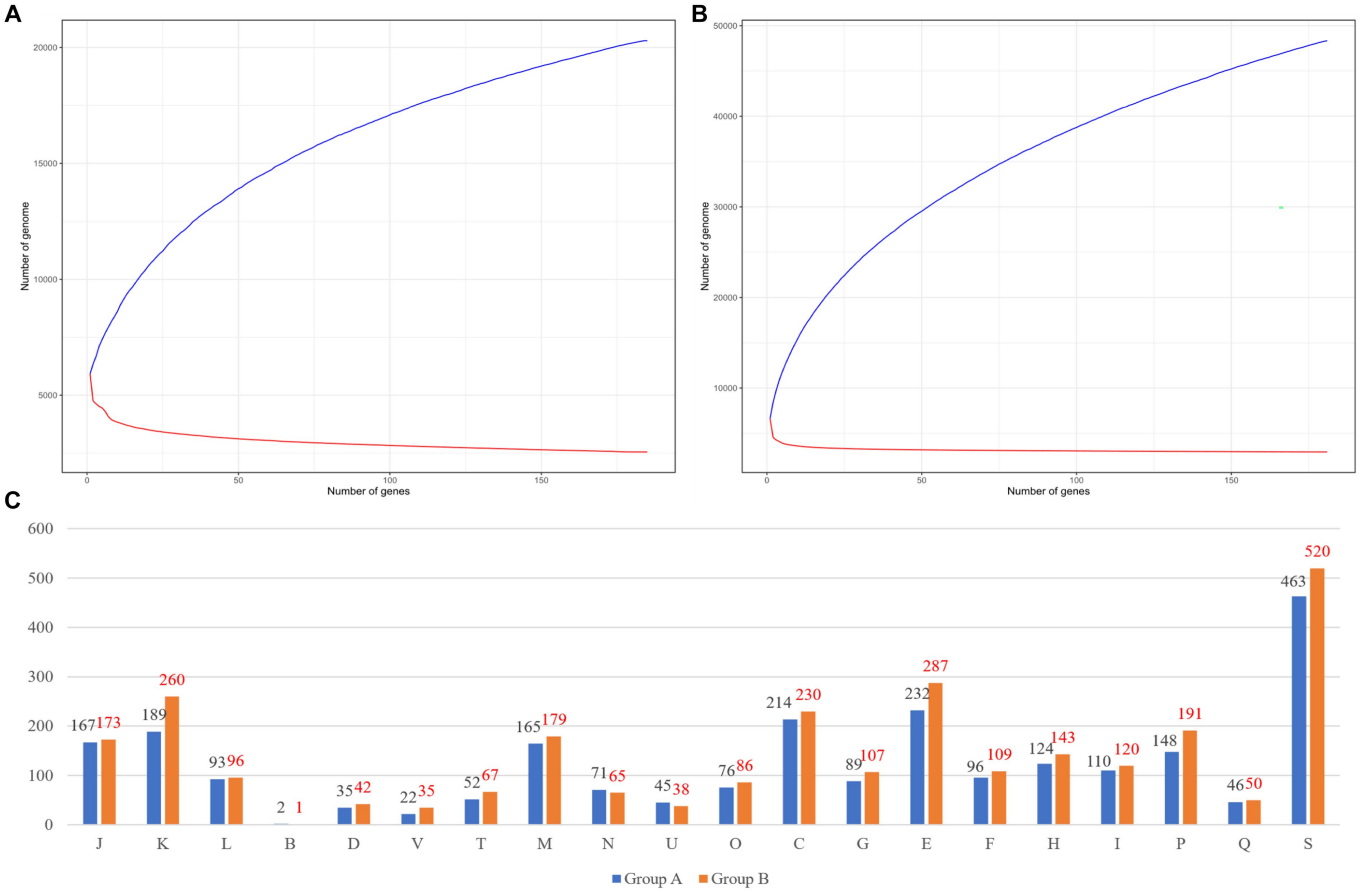
Pan-genome results of each group. **(A)** Group A’s pan-genome and core-genome results. **(B)** Group B’s pan-genome and core-genome results. **(C)** Differences between group A and group B. The number of COGs in group A is indicated by a black color and the number of COGs in group B is indicated by a red color. COGs categories are as follows: J, translation, ribosomal structure and biogenesis; (A), RNA processing and modification; (K), transcription; (L), replication, recombination, and repair; (B), chromatin structure and dynamics; D, cell cycle control, cell division, and chromosome partitioning; Y, nuclear structure; V, defense mechanisms; T, signal transduction mechanisms; (M), cell wall/membrane/envelope biogenesis; (N), cell motility; (Z), cytoskeleton; (W), extracellular structures; (U), intracellular trafficking, secretion, and vesicular transport; O, posttranslational modification, protein turnover, and chaperones; (X), mobilome, prophages, and transposons; C, energy production and conversion; G, carbohydrate transport and metabolism; E, amino acid transport and metabolism; F, nucleotide transport and metabolism; H, coenzyme transport and metabolism; I, lipid transport and metabolism; P, inorganic ion transport and metabolism; Q, secondary metabolites biosynthesis, transport, and catabolism; R, general function prediction only; and S, function unknown.

We compared genomic patterns among 366 species of *Burkholderia* which were collected from NCBI. Prokka analysis was performed using species of *Burkholderia*, while pan-genome and core-genome analyses were performed using PEPPAN with the Prokka output (Figure 3). In group A (*n* = 185) we observed, 14,066 cloud genes, 2,465 shell genes, 682 soft-core genes, and 2,553 strict-core genes (Figure 3A). The pan-genome results of group A of *Burkholderia* showed 18.53% of total core genes. Group A was confirmed to have 5,770 genes per genome with 20,292 pan-genes and 2,553 core-genes. In group A, Heaps’ law showed a Gamma value of 0.289 ± 0.002 and a Kappa value of 4493.245 ± 46.171. In the Power law model, an Alpha value of 0.638 ± 0.013 and a Kappa value of 964.425 ± 32.586 were observed. The power law model for core genomes showed an Alpha value of 0.149 ± 0.001 and a Kappa value of 5591.983 ± 20.623. In the results for group B (*n* = 181), 39,867 cloud genes, 4,986 shell genes, 324 soft-core genes, 222 core genes, and 2,949 strict-core genes were observed (Figure 3B). Ran-genome results for group B of *Burkholderia* showed 7.22% of total core genes. In group B, Heaps’ law showed a Gamma value of 0.389 ± 0.001 and a Kappa value of 6444.550 ± 30.636. In the Power law model, an Alpha value of 0.629 ± 0.003 and a Kappa value of 2719.814 ± 21.208 were observed. The power law model for core genomes showed an Alpha value of 0.099 ± 0.007 and a Kappa value of 4795.235 ± 131.235. Strict core genes showed 100% similarity, while core genes showed similarities ranging from over 99% to less than 100%. Soft-core genes showed similarities ranging from over 95% to less than 99%. Genes with similarities ranging from 0% to 15% were classified as cloud genes. Those with similarities ranging from 15% to 95% were categorized as shell genes. Pangenomes in both groups showed Alpha values under one. The pan-genome analysis of *Burkholderia* began in 2009, where 56 species of *Burkholderia* were found to possess 4,000 genes, with a core-genome consisting of 1,000 genes (Ussery et al., 2009). Subsequently, Bochkareva et al. (2018) analyzed 127 *Burkholderia* species using the micropan tool in the R environment. They conducted an analysis employing the binomial mixture model and Chao’s lower bound. In this study, we attempted to conduct pan-genome analysis using the micropan tool, similar to previous research methods. However, we were unable to complete the analysis due to insufficient computer performance. Because of the large number of samples, we divided our samples into group A and group B based on ANI results. For pan-genome analysis, we utilized PEPPAN, which employs representative genes obtained through clustering to expedite the analysis process. When comparing our research findings to those of Bach et al. (2022a), we observed that group B, which included BCC, showed a slightly higher number of 2,949 strict core genes compared to ROARY results.

Ortholog analyses of core genes in group A and group B were performed using eggNOG. We visualized COGs. We analyzed functional annotations for 2,553 core-genes from group A. We visualized the results of COGs with both group A and group B (Figure 3C). Most differences were shown in V (37%) and K (27%), respectively. Pan-genome analyses have been steadily conducted for diverse *Burkholderia* species based on genomics of *B. contaminans* (Kim et al., 2023) or *B. cepacia* (Ahmad and Azam, 2020; which showed similar results with ours) and BCC classification (Jin et al., 2020). Recently, Bach et al. (2022b) reported a pan-genome focused study that analyzed not only BCC but also *B. sensu lato* and *B. sensu stricto*. Through pan-genome analysis, Lood et al. (2021) confirmed patient diversity using genomics. The present study has identified unique attributes exclusive to *Burkholderia* using a pattern analysis of BGCs based on a comparison between BCC and BPC of *Burkholderia*. Our results provide unique insights and can be used to perform further genome analysis for pathogenic *Burkholderia*.

### 3.4 Analysis of BGC

**Figure 4:**
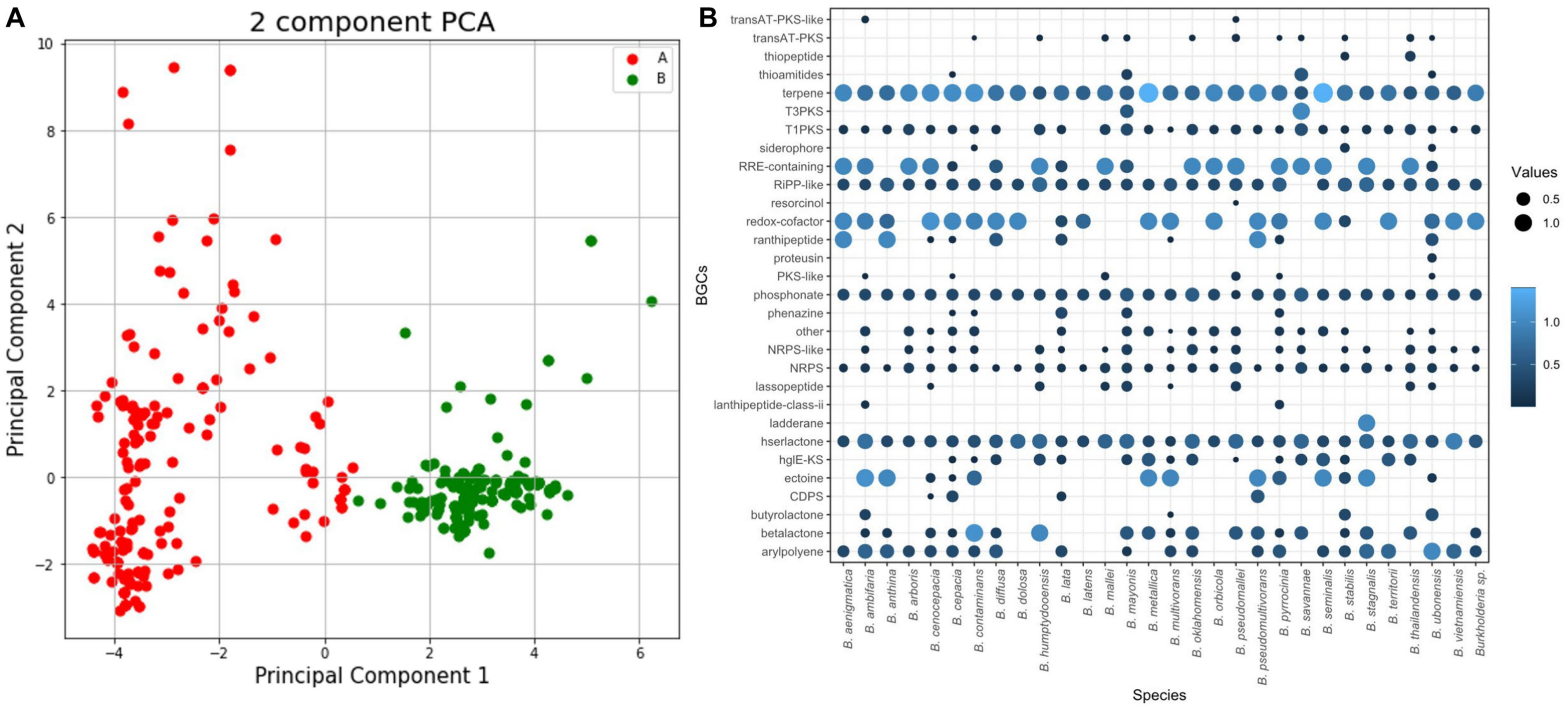
BGC analysis for 366 *Burkholderia* spp. **(A)** AntiSMASH results of *Burkholderia* spp. We visualized BGCs in each species and combined similar BGCs in one group. **(B)** PCA analysis using antiSMASH results matrix and color by groups.

Using antiSMASH, this study detected and categorized 6,666 BGCs belonging to 113 kinds of BGCs and 30 classes including polyketide synthase (PKS), terpene, and siderophore. We analyzed BGCs using PCA (Figure 4A). During the process of visualizing PCA results, we examined both environmental and pathological samples. However, no significant differences were observed. Instead, PCA results revealed a more distinct separation based on species and groups (data not shown).

When visualizing the results of PCA for each species, we observed distinct clusters corresponding to *B. pseudomallei* and *B. mallei*. *B. pseudomallei* was the most frequently analyzed species with 128 samples, which might have influenced PCA results, showing a distinct clustering. Despite the high similarity observed between *B. pseudomallei* and *B. mallei* in ANI results, they exhibited clear separation in the PCA analysis (Supplementary Figure 2). Investigation of 366 *Burkholderia* strains revealed an average of 17 BGCs per strain. *B. latens* AU0505 was found to possess 7 BGCs, while *B. pseudomallei* 1710b, *B. pseudomallei* BSR, *B. pseudomallei* 406e, and *B. mayonis* BDU8 were found to harbor 26 BGCs each. In this study, we calculated the number of BGCs in each species. BCC and BPC showed higher numbers of candidate BGCs than previously known BGCs (Mullins and Mahenthiralingam, 2021; Figure 4B). We found three novel BGCs from *B. cenocepacia* J2315, *B. pseudomallei* 3,000,047,530, and *B. pyrrocinia* MS455. A study on genomic diversity and metabolic capabilities of *B. sensu lato*, has found that *Burkholderia* possesses a more diverse set of BGCs than other genera (Mullins and Mahenthiralingam, 2021; Petrova and Mahenthiralingam, 2022). Alam et al. (2021) have also confirmed genome mining of BGCs in *Burkholderia* including *B. latens*, *B. cenocepacia*, *B. cepacia*, *B. ambifaria*, and *B. lata*. Our study showed similar patterns of BGC possession and confirmed novel species recently assigned to BCC and BPC.

**Figure 5:**
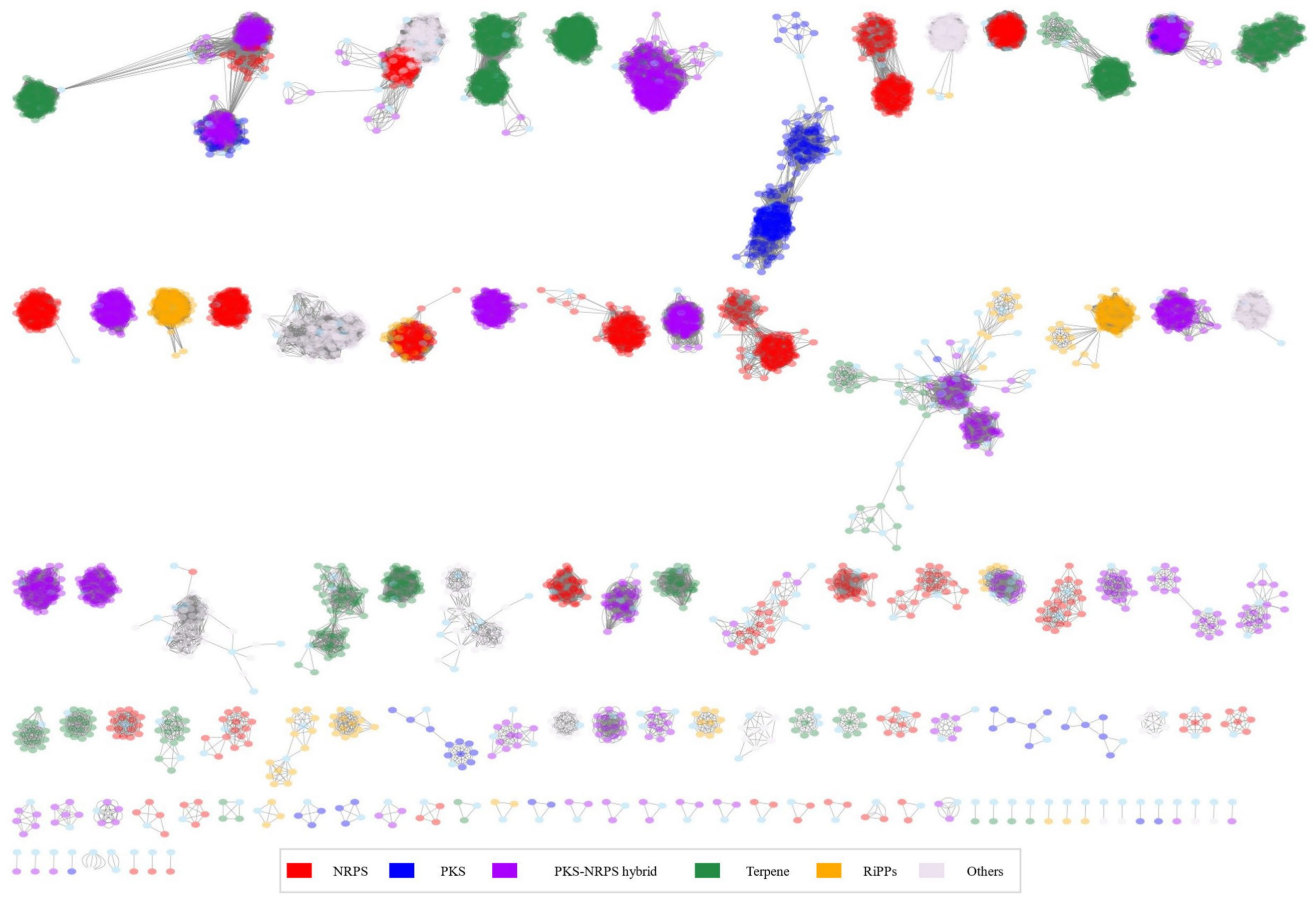
Network analysis for 366 *Burkholderia* spp.

Based on these results, we hypothesized that there would be distinct metabolite patterns depending on the species of *Burkholderia*. BiG-SCAPE results were then subjected to network analysis, categorizing antiSMASH outcomes into NRPS, terpene, PKSI, RiPPs, PKSother, PKS-NRPS hybrids, and others (Figure 5; Supplementary Table 5). A total of 192 types of BGCs were found and compared with the MiBIG database (Supplementary Tables 6, 7). Two types of alkaloids, 48 types of NRPS (including one kind of NRPS-alkaloid and 13 kinds of NRPS-polyketide BGCs), 30 kinds of polyketide, 28 kinds of RiPP, 12 kinds of saccharide, 17 kinds of terpene, and 55 BGCs of others were found. We confirmed the most popular 667 terpene-related BGCs known as carotenoids-related BGCs from *Myxococcus xanthus*. We found 44 novel BGCs in distinct samples. These novel BGCs contained 13 other BGCs classes and followed polyketide. For each BGC, known compounds were compiled and core gene information was organized to detect gene clusters (Table 2; Liu and Cheng, 2014; Kunakom and Eustáquio, 2019) and undertake a more detailed analysis, described below.

**Table 2:** Secondary metabolites reported from *Burkholderia.*

BGCs classification	Compounds	Genes	References
NRPS	Fragin	*hamA-G*	Jenul et al. (2018)
NRPS	Glidopeptin A	*glpCDE*	Wang et al. (2018)
NRPS	Sulfazecin	*sulC-K, sulM*	Li et al. (2017)
NRPS	Valdiazen	*hamACDEG*	Liu and Cheng (2014)
PKS-NRPS	Bactobolin	*btaKNOML*	Esmaeel et al. (2018)
PKS-NRPS	Burkholdin	*bksABCDEFG*	Esmaeel et al. (2018)
PKS-NRPS	fk228	*depABCDE*	Gong et al. (2023)
PKS-NRPS	Glidobactin A	*glbFC*	Esmaeel et al. (2018)
PKS-NRPS	Malleilactone	*malAF*	Esmaeel et al. (2018)
PKS-NRPS	Occidiofungins	*ocfA-N*	Gu et al. (2011)
PKS-NRPS	Spiruchostatin	*spiABC1DE1C2E2*	Gong et al. (2023)
PKS-NRPS	Spliceostatins	*fr9CDEFGHI*	Eustáquio et al. (2014)
PKS-NRPS	Thailandamides	*thaA-R*	Ishida et al. (2010)
PKS-NRPS	Thailandepsins/Burkholdacs	*tdpABC1DE1C2E2*	Esmaeel et al. (2018)
PKS-NRPS	Thailanstatins	*tstCDEFGHI*	Esmaeel et al. (2018)
trans-AT-PKS	Thailandene A-C	*orgA-M*	Park et al. (2020)
RiPP	Capistruin	*capABCD*	Knappe et al. (2009)
RiPP	Cepacin A-B	*ccnA-P*	Mullins et al. (2019)
RiPP	Rhamnolipids	*rhlABC*	Dubeau et al. (2009)
RiPP	Ubonodin (lasso peptide)	*uboABCD*	Cheung-Lee et al. (2020)
Other	Hydrogen cyanide	*hcnABC*	Ryall et al. (2008)
Other	Phenazines (PCA/phencomycins)	*phzABCDEFIR*	Hendry et al. (2021)
Other	Phenylpyrrole (pyrrolnitrin)	*prnABCD*	Hammer et al. (1999)
Other	Quinolone (burkholone, pseudane)	*hhqABCDEFG, hmqABCDEFG, pqsABCDE*	Prothiwa et al. (2021)
Other	Tropolonea	*troR1, troK, troR2*	Wang et al. (2016)

### 3.5 Pattern analysis for NRPS and PKS

**Figure 6:**
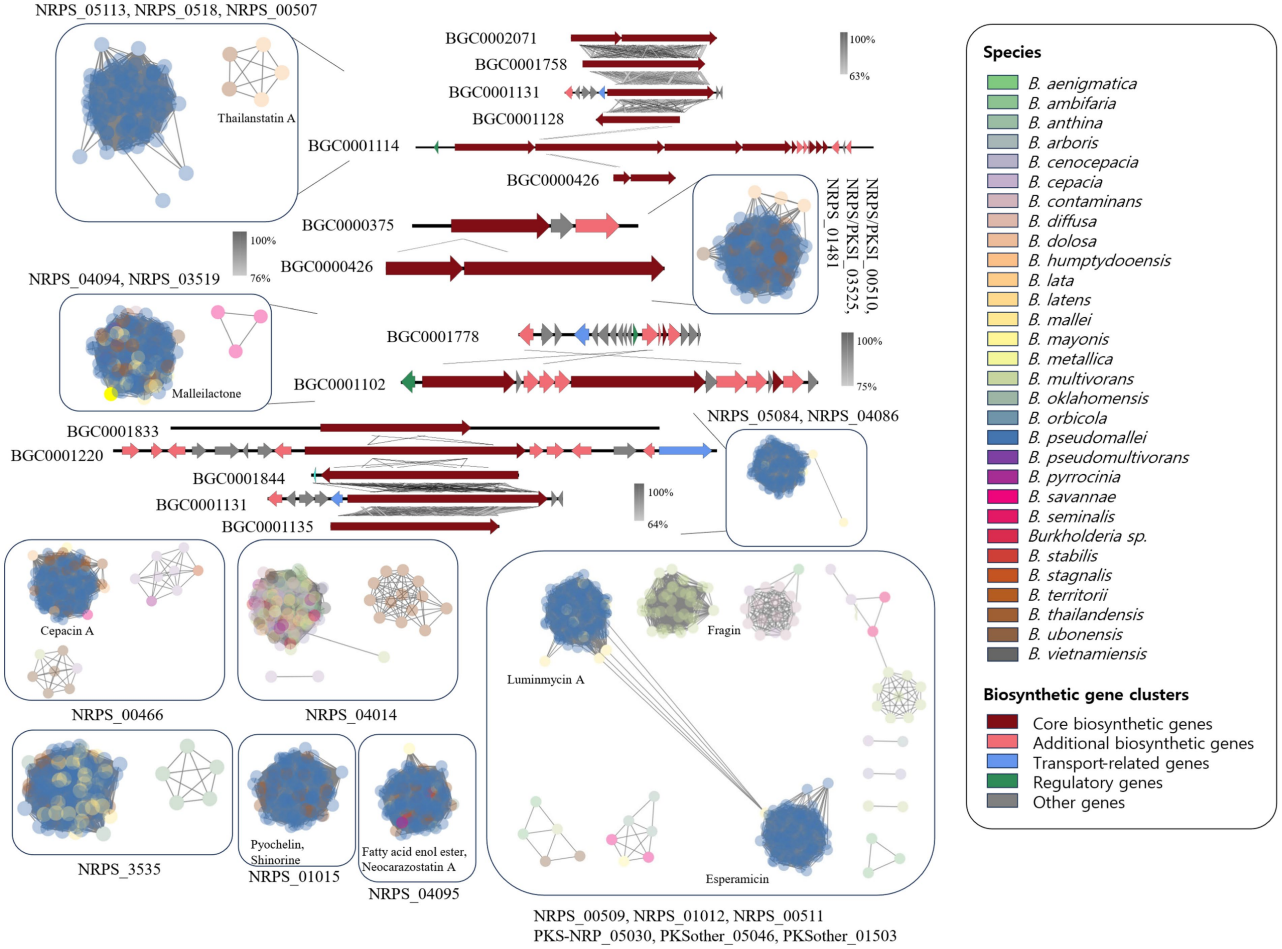
Network analysis of NRPS and PKS related clan and visualization of reference BGCs.

The NRPS network yielded 2,345 BGCs with 138,894 links and 118 families, including 28 singletons. Many compounds have been reported in *Burkholderia*. The following compounds were identified in NRPS gene clusters: valdiazen, glidopeptin A, rhizomide A, occidiofungins, fragin, sulfazecin, and icosalide A/B. PKSI network yielded 459 BGCs with 22,730 links, and 30 families, including 7 singletons. PKSother network showed 642 BGCs, 33,515 links, 48 families, and 15 singletons. The network of PKS-NRPS hybrids yielded 524 BGCs with 29,440 links and 31 families, including 6 singletons. PKS-NRPS hybrid BGCs could be categorized as follows based on their gene cluster types: NRPS-T1PKS, PKS-NRPS, and transAT-PKS. Network analysis was visualized with reference gene clusters (Figure 6). We found 44 types of BGCs, and BGCs were known from *Burkholderia, Paraburkholderia,* and *Pseudomonas*. In the network analysis, we visualized the node as the sample species and the edge as the link between samples. Each node color indicates a species. We visualized the reference for BGC from the MiBIG database in each dependent network. We grouped each clan based on the results of BiG-SCAPE. In the results of network analysis, some BGCs were calculated in the part of PKS-NRPS hybrid, NRPS, and PKSother. We visualized the main clans in NRPS, PKS-NRPS hybrid, PKSI, and PKSother. We also visualized BGCs known as *Burkholderia*-related species such as *Paraburkholderia* and *Pseudomonas*. Although network analyses of BGCs through genome mining and broader global analyses have been reported in previous studies, only *B. ambifaria* has been found to focus on the secondary metabolite (Mullins et al., 2019).

Clan members within NRPS_05113 were confirmed to include BGC0002071, BGC0001758, BGC0001131, and BGC0001128, which are known to generate virginiafactin, rhizomide A, ambactin, and luminmide, respectively. The similarity between each BGC was visualized using Easyfig. Reference BGCs identified in each network were compared and visualized as shown in Figure 6. The representative compounds identified in each clan are also indicated. Although the comparison of BGCs between BCC and the BPC was based on gene cluster similarity analysis and network analysis, a greater diversity of BGCs was observed than anticipated. Similar BGCs were also identified in other plant species, fungi, and beyond *Burkholderia*. These unique BGC-specific features across different species have expanded the scope of *Burkholderia* research, enabling more extensive investigations. Most clans revealed that *B. pseudomallei* was the main species excluding NRPS_04014. The NRPS_04014 clan contained ornibactin and indigoidine-related BGCs. Ornibactin was known as a siderophore from *B. cenocepacia*. In 44 types of BGCs, we confirmed several types of siderophore. Siderophore-related BGCs were confirmed. Ornibactin are known to possess *Pseudomonas* and siderophore-like compounds (Anthoni et al., 1995).

### 3.6 Identification of siderophore and pattern analysis for BCC and BPC

We predicted that complex and/or species would have different types of siderophore-related BGCs. To test our hypothesis, we performed additional analysis. Siderophores have been reported to exist in BCC. A comparative transcriptome study of *B. pseudomallei* reported on the induction of siderophores that were able to adapt and survive in the host (Ghazali et al., 2023). However, in antiSMASH results, species belonging to group B did not show the presence of siderophore-related clans. Since *Burkholderia* are known to produce various siderophores that might not be detected by antiSMASH, we conducted further analysis using the results of MiBIG similarity to explore siderophore gene clusters (Table 3). In *Burkholderia*, the following types of siderophores have been reported: ornibactin, malleobactin, cepaciachelin, pyochelin, and cepabactin (Butt and Thomas, 2017). For ornibactin biosynthesis, NRPS genes such as *orbI* and *orbJ* are known, and we used them to search for the corresponding BGCs (Agnoli et al., 2006). Pyochelin biosynthesis involves *pchE* and *pchF* known as NRPS (Quadri et al., 1999). We used these genes to search for the corresponding BGCs with malleobactin related genes as well (Alice et al., 2006). To find cepaciachelin, we collected genes such as *cphA*, *cphB*, and *cphC* (Esmaeel et al., 2016). Cepabactin-related genes have not been described. Malleonitrone is a compound formed by the combination of malleobactins and pyochelin (Trottmann et al., 2019). Other siderophores, burkholdac A and pseudomonine, were identified in group A, which belonged to the BPC, but not in group B. Burkholdac A was exclusively found in *B. savannae* and *B. thailandensis*, while pseudomonine was detected in *B. humptydooensis*, *B. mayonis*, *B. pseudomallei*, and *B. thailandensis* only. Siderophores are known to be species-specific. Our investigation revealed that species within the same complex in *Burkholderia* commonly possessed siderophore-related BGCs (Sandy and Butler, 2009). Alongside siderophores, compounds related to terpenes and RiPPs have been reported. Additionally, certain strains known as plant pathogens, such as *B. glumae*, have been reported to contain a variety of secondary metabolites. For instance, toxoflavin (Kim et al., 2004), gladiofungin A (gladiostatin; Niehs et al., 2020), and gladiolin from *B. gladioli* (Song et al., 2017) have been identified. Furthermore, compounds such as enacyloxin, which has not been confirmed due to limited known genes, have been reported in *B. ambifaria.* They are believed to contain PKS-related modules (Mahenthiralingam et al., 2011).

**Table 3:** Pattern of siderophore in each *Burkholderia* species.

Species/number of samples		Malleilactone	Pyrrolnitrin	Cepacin A	Burkholdac A	Ornibactin	Enterobactin	Pseudomonine	Pyochelin	Pyocyanine
*B. aenigmatica*	(*n* = 1)	0	0	0	0	1	0	0	0	0
*B. ambifaria*	(*n* = 8)	0	0	0	0	11	0	0	1	0
*B. anthina*	(*n* = 2)	0	0	0	0	3	0	0	0	0
*B. arboris*	(*n* = 1)	0	0	0	0	1	0	0	1	0
*B. cenocepacia*	(*n* = 27)	0	1	0	0	29	2	0	24	0
*B. cepacia*	(*n* = 20)	0	1	0	0	20	0	0	17	3
*B. contaminans*	(*n* = 14)	1	0	0	0	15	0	0	9	0
*B. diffusa*	(*n* = 1)	0	0	0	0	2	0	0	0	0
*B. dolosa*	(*n* = 5)	0	0	0	0	5	0	0	0	0
*B. humptydooensis*	(*n* = 1)	0	0	0	0	0	0	3	0	0
*B. lata*	(*n* = 3)	0	0	0	0	3	0	0	2	0
*B. latens*	(*n* = 3)	0	0	0	0	3	0	0	0	0
*B. mallei*	(*n* = 27)	14	0	0	0	0	0	0	0	0
*B. mayonis*	(*n* = 2)	2	0	0	0	0	0	2	0	0
*B. metallica*	(*n* = 1)	0	0	0	0	1	0	0	0	0
*B. multivorans*	(*n* = 50)	1	0	0	0	49	0	0	0	0
*B. oklahomensis*	(*n* = 3)	3	0	2	0	0	0	0	0	0
*B. orbicola*	(*n* = 1)	0	0	0	0	1	0	0	1	0
*B. pseudomallei*	(*n* = 128)	117	7	120	0	1	0	1	126	0
*B. pseudomultivorans*	(*n* = 1)	0	0	0	0	1	0	0	0	0
*B. pyrrocinia*	(*n* = 5)	0	2	0	0	7	0	0	0	0
*B. savannae*	(*n* = 2)	3	2	0	3	3	0	0	0	0
*B. seminalis*	(*n* = 2)	0	0	0	0	2	0	0	2	0
*Burkholderia* sp.	(*n* = 18)	0	0	0	0	2	0	0	0	0
*B. stabilis*	(*n* = 2)	0	0	0	0	3	0	0	3	0
*B. stagnalis*	(*n* = 1)	0	0	0	0	1	0	0	0	0
*B. territorii*	(*n* = 1)	0	0	0	0	1	0	0	0	0
*B. thailandensis*	(*n* = 19)	17	0	5	3	0	0	12	17	0
*B. ubonensis*	(*n* = 7)	2	3	0	0	7	0	0	0	1
*B. vietnamiensis*	(*n* = 10)	0	0	0	0	11	0	0	0	0

**Figure 7:**
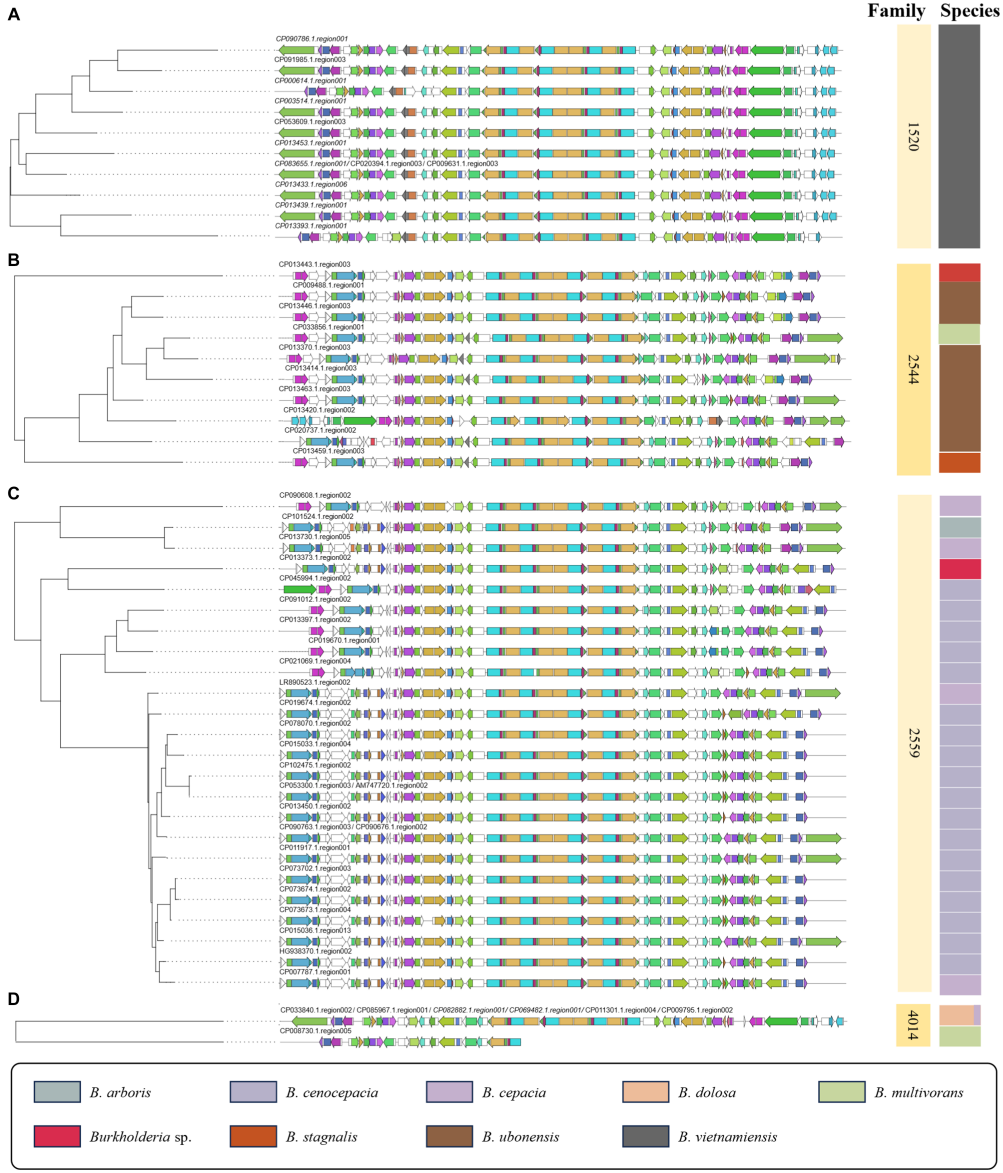
Visualization of ornibactin-related biosynthetic genes in NRPS_4602 clan. **(A)** The phylogeny of 1520 family. **(B)** The phylogeny of 2544 family. **(C)** The phylogeny of 2559 family. **(D)** The phylogeny of 4014 family.

This study utilized ClusterBlast to extract similar gene clusters. We structured them into a table using Python. Among confirmed siderophore-related compounds, we identified nine, namely malleilactone, pyrrolnitrin, cepacin A, malleobactin, burkholdac A, ornibactin, enterobactin, pseudomonine, pyochelin, quinolobactin, and pyocyanine. Among these, ornibactin was the most commonly found across various species. The NRPS_4602 clan, which harbored the ornibactin BGC, encompassed gene clusters for ornibactin, pyocyanine, indigoidine, burkholderic acid, anabaenopeptin, burkholdac A, depudecin, and fellutamide B. We visualized ornibactin BGCs within this clan using CORASON (Figure 7). Within the NRPS_4602 clan, ornibactin BGCs were distributed across families FAM_1520, FAM_2544, FAM_2559, FAM_4014, FAM_4564, FAM_4602, and FAM_5592. Each family’s gene clusters exhibited similarity, further confirming their species-specific nature.

In the case of FAM_1520, it mainly corresponded to ornibactin BGCs identified in *B. vietnamiensis*, while FAM_2544 was primarily associated with *B. ubonensis*. For all other families, we visualized gene clusters as shown in Figure 7 and Supplementary Figures 2, 3. The observed similarity in gene clusters within the same species suggested their potential utility in distinguishing between BCC and BPC. Previous studies on siderophores have been limited to a small number of *Burkholderia* strains (Esmaeel et al., 2016). Our research presents a new direction for the genome mining of the secondary metabolism, with an expanded sample size and an analytical approach.

## 4 Conclusion

To date, *Burkholderia* has been known for its diversity in species, but more recent reclassification efforts have associated all complexes within the *Burkholderia* genus as having pathogenic characteristics. In this study, we aimed to analyze features of *Burkholderia* through ANI analysis and reference genes, categorizing them into BCC and BPC for analysis. Through PCA analysis based on antiSMASH results, BCC and BPC were revealed to be distinct. This indicates that each complex is likely to possess a different pattern of BGCs. The network analysis, BiG-SCAPE analysis, and comparative analysis using the MiBIG database revealed that BGCs differed by complex. Through network analysis and visualization of siderophores specific to each species, we also demonstrated unique siderophore patterns for each species and/or complex. Furthermore, this study explored known BGCs reported not only in *Burkholderia*, but also in *Pseudomonas* and *Paraburkholderia*. By visualizing the gene cluster of ornibactin, the siderophore found in the highest number of species, we anticipate that pattern analysis could be further advanced from a broader perspective. This research became possible due to the increasing number of *Burkholderia* genomes and the identification of various BGCs in *Burkholderia*. However, identifying novel BGCs remains challenging. We could only confirm that results for just three BGCs were not detected from the known MiBIG database. This study serves as a comprehensive investigation into NRPS and PKS. It contributes to future research on secondary metabolites in *Burkholderia*.

## Author contributions

BK: Conceptualization, Data curation, Formal analysis, Investigation, Methodology, Project administration, Software, Validation, Writing – original draft, Writing – review & editing. S-RH: Data curation, Methodology, Software, Writing – original draft, Writing – review & editing. HL: Formal analysis, Methodology, Software, Writing – original draft, Writing – review & editing. T-JO: Conceptualization, Funding acquisition, Investigation, Project administration, Resources, Supervision, Validation, Writing – original draft, Writing – review & editing.
