# Erratum: Insights into group-specific pattern of secondary metabolite gene cluster in *Burkholderia* genus

**DOI:** 10.3389/fmicb.2024.1469596

**Published:** 2024-08-09

**Authors:** Frontiers Production Office

**Affiliations:** Frontiers Media SA, Lausanne, Switzerland

**Keywords:** *Burkholderia* genus, comparative genomics, biosynthetic gene cluster, pattern analysis, network analysis

Due to an editorial error, the article type was incorrectly given as “Review” when it is an “Original Research” article.

The publisher apologizes for this mistake. The original article has been updated.

