# Supplementary material for: Insights into group-specific pattern of secondary metabolite gene cluster in Burkholderia genus
Source: Front Microbiol. 2024 Jan 16;14:1302236. doi: 10.3389/fmicb.2023.1302236 (PMC10826400; doi:10.3389/fmicb.2023.1302236)
Supplement: Supplementary file 1 [file Data_Sheet_1.DOCX]

Supplementary Material

Insights into group-specific pattern of secondary metabolite gene cluster in *Burkholderia* genus

Byeollee Kim^1^, So-Ra Han^2^, Hyun Lee^2,3^, Tae-Jin Oh^1,2,4*^

^1^ Department of Life Science and Biochemical Engineering, Graduate School, SunMoon University, Asan 31460, Republic of Korea

^2^ Genome-based BioIT Convergence Institute, Asan 31460, Republic of Korea

^3^ Division of Computer Science and Engineering, SunMoon University, Asan 31460, Republic of Korea

^4^ Department of Pharmaceutical Engineering and Biotechnology, SunMoon University, Asan 31460, Republic of Korea

*** Correspondence:** Tae-Jin Oh; [tjoh3782@sunmoon.ac.kr](mailto:tjoh3782@sunmoon.ac.kr)

# Supplementary Data

To genome mining of *Burkholderia*, we exclude duplicate sample, over 5 contamination value, and under 80 % similarity in ANI results (Supplementary Table 1). We organized our 366 *Burkholderia* dataset (Supplementary Table 2). We reclassified *Burkholderia* sp. samples if they shown high similarity with other samples, we listed in Supplementary Table 3. We progressed MLST analysis using blast with curated gene as the database, and we visualized using grapetree using blast results transforms matrix. Curated genes were confirmed in BCC and *B. pseudomallei*. But we cannot find any significant differences (Supplementary Figure 1). Besides, we confirmed taxonomic difference using known environmental genes and pathological genes (Supplementary Table 4). We visualized each species in PCA. *Burkholderia pseudomallei* and *Burkholderia mallei* have well gathered, but several species exist in similar place (Supplementary Figure 2). We organized our all the results from antiSMASH in Supplementary Table 5. For the pattern analysis, we analysis network using BiG-SCAPE. All the cluster’s distance and Jaccard index were organized in Supplementary Table 6. In siderophore pattern analysis, we selected ornibactin which possess several kinds of species. To make understanding visualization, we divided by group of NRPS family that BiG-SCAPE results categorized similar gene cluster as calculated by network analysis. In Supplementary Figures 3 and 4, we visualized gene cluster in each ornibactin-related BGCs and shown each species as the color.

# Supplementary Figures


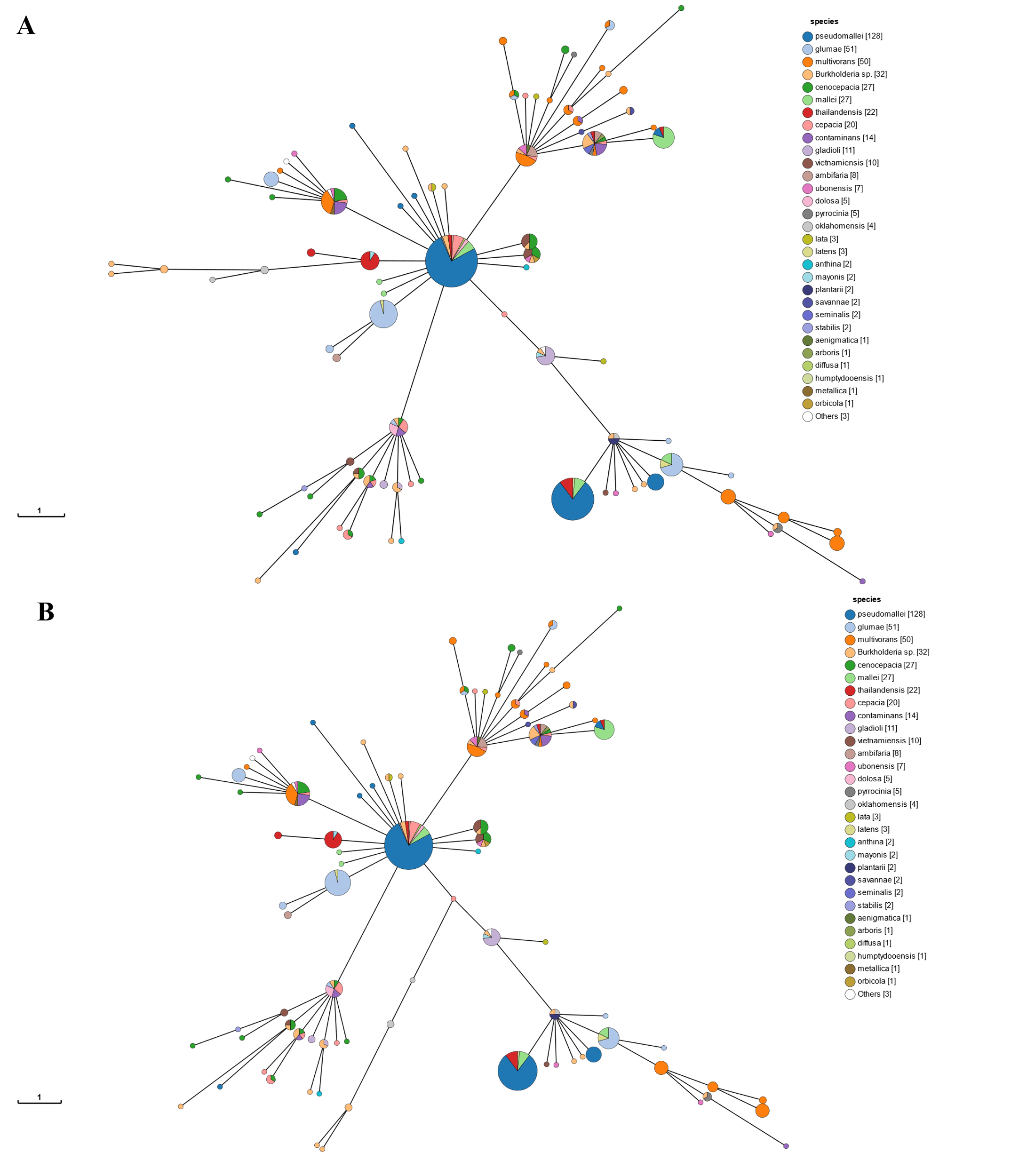


Supplementary Figure 1. The visualization of MLST. (A) The visualization of MLST in BCC using grapetree. We found that BCC’s curated genes from PubMLST which included *aptD*, *gltB*, *gyrB*, *recA*, *lepA*, *phaC*, and *trpB*. (B) The visualization of MLST in *Burkholderia pseudomallei* using curated genes such as *ace*, *gltB*, *gmhD*, *lepA*, *lipA*, *narK*, and *ndh*.

**
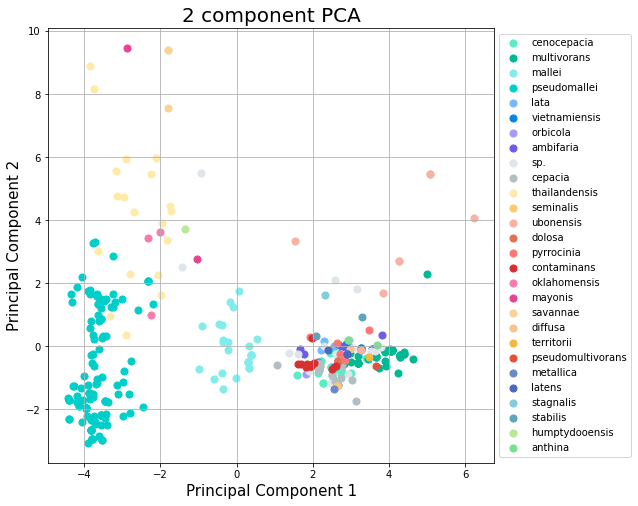
**

Supplementary Figure 2. The principal component analysis. Each point represented sample and color by species.


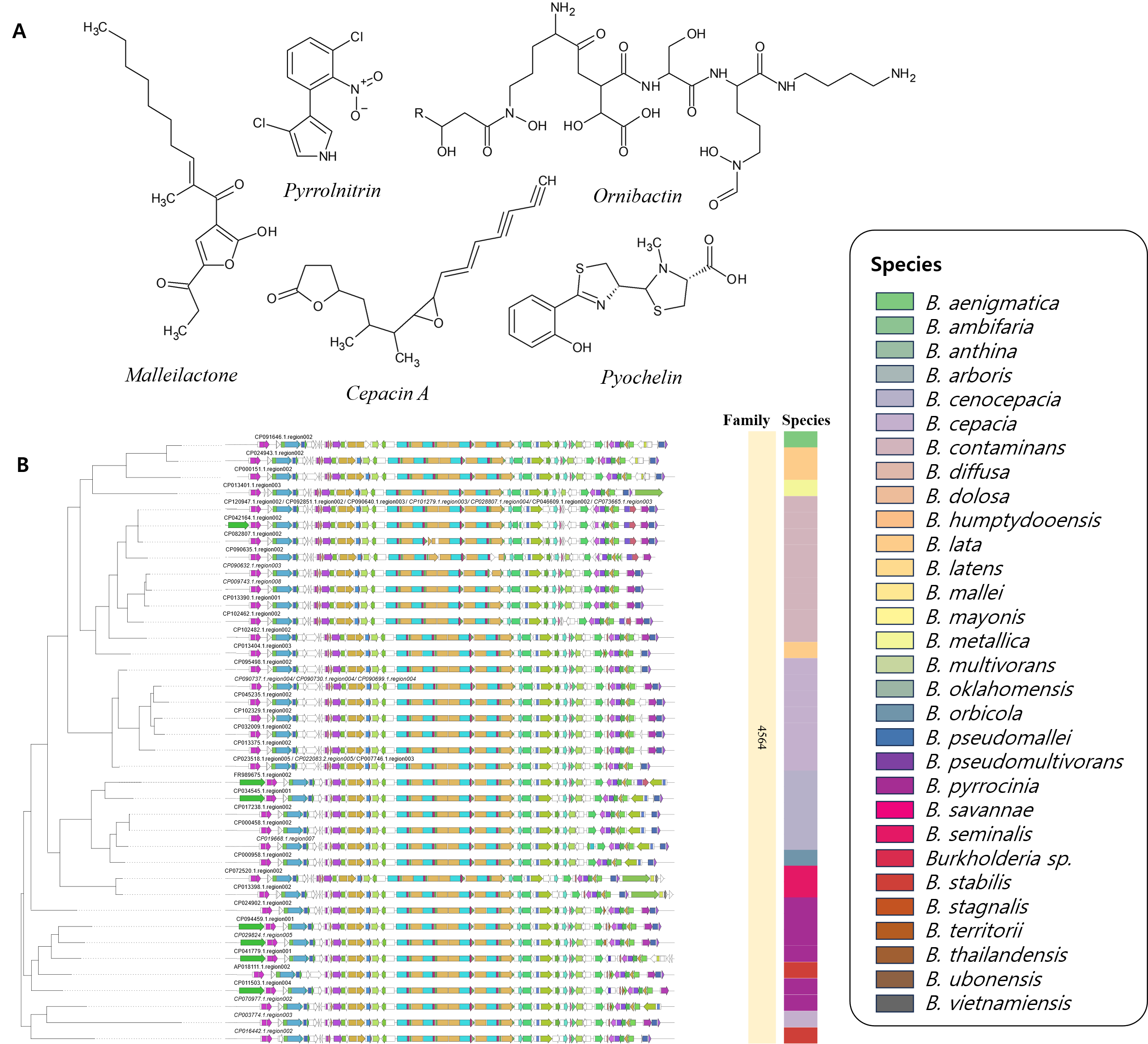


Supplementary Figure 3. The siderophore chemical structures (A) and visualization of ornibactin-related gene clusters (B) in NRPS_family_4564.


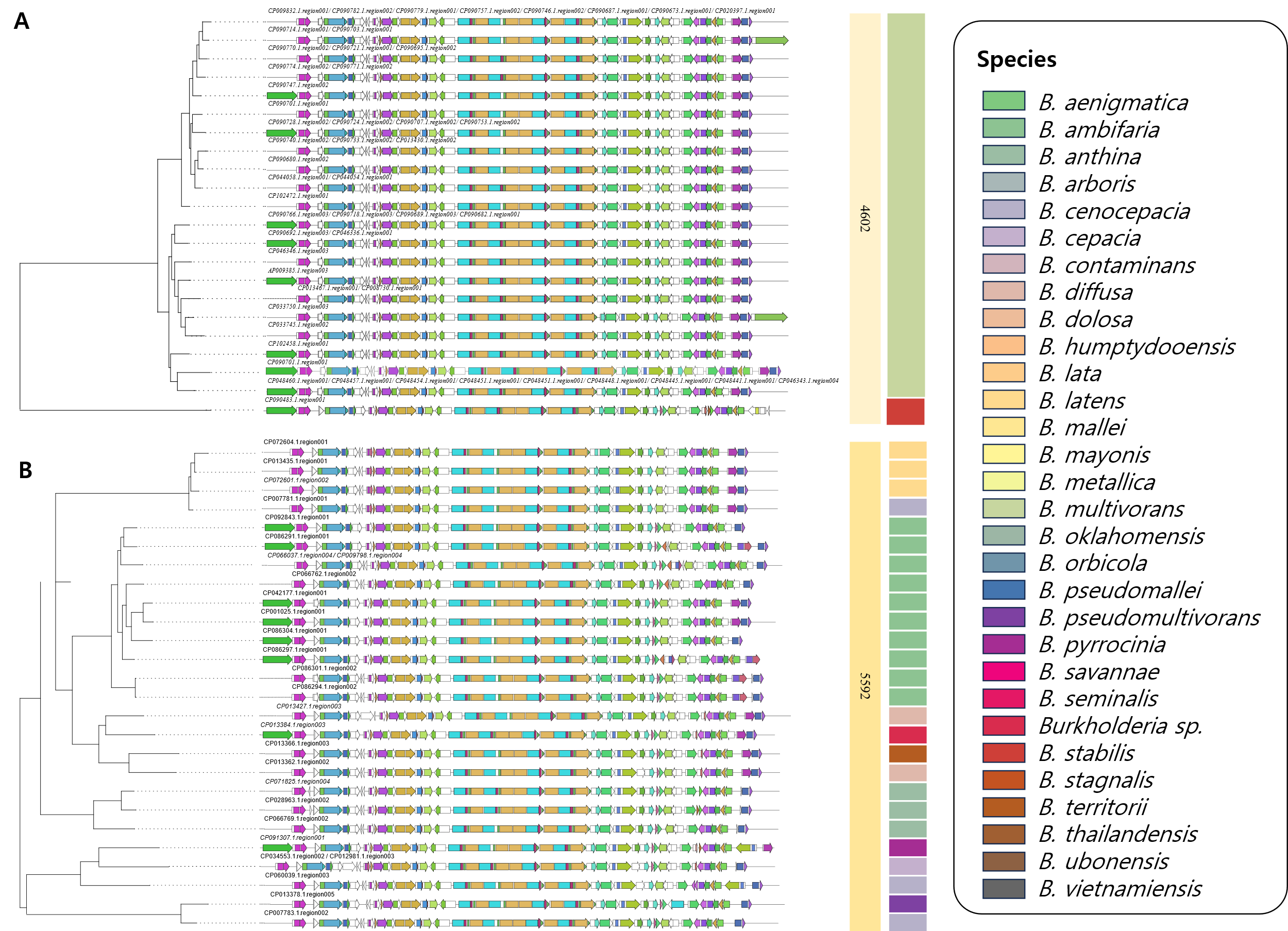


Supplementary Figure 4. The visualization of ornibactin-related gene cluster in NRPS_family_4602 (A) and NRPS_family_5592 (B).
